# Have (*R*)-[^11^C]PK11195 challengers fulfilled the promise? A scoping review of clinical TSPO PET studies

**DOI:** 10.1007/s00259-021-05425-w

**Published:** 2021-08-13

**Authors:** Fabien Chauveau, Guillaume Becker, Hervé Boutin

**Affiliations:** 1 University of Lyon, Lyon Neuroscience Research Center (CRNL), CNRS UMR5292, INSERM U1028, University Lyon 1, Lyon, France; 2 GIGA – CRC In Vivo Imaging, University Liege, Liege, Belgium; 3 University of Lyon, CarMeN Laboratory, INSERM U1060, University Lyon 1, Hospices Civils Lyon, Lyon, France; 4 Faculty of Biology Medicine and Health, Wolfson Molecular Imaging Centre, University of Manchester, Manchester, UK; 5 Wolfson Molecular Imaging Centre, University of Manchester, Manchester, UK; 6 Geoffrey Jefferson Brain Research Centre, Manchester Academic Health Science Centre, Northern Care Alliance & University of Manchester, Manchester, UK

**Keywords:** Translocator protein 18 kDa (TSPO), Clinical PET imaging, (*R*)-[^11^C]PK11195, Radiotracer, Neuroinflammation

## Abstract

**Purpose:**

The prototypical TSPO radiotracer (*R*)-[^11^C]PK11195 has been used in humans for more than thirty years to visualize neuroinflammation in several pathologies. Alternative radiotracers have been developed to improve signal-to-noise ratio and started to be tested clinically in 2008. Here we examined the scientific value of these “(*R*)-[^11^C]PK11195 challengers” in clinical research to determine if they could supersede (*R*)-[^11^C]PK11195.

**Methods:**

A systematic MEDLINE (PubMed) search was performed (up to end of year 2020) to extract publications reporting TSPO PET in patients with identified pathologies, excluding studies in healthy subjects and methodological studies.

**Results:**

Of the 288 publications selected, 152 used 13 challengers, and 142 used (*R*)-[^11^C]PK11195. Over the last 20 years, the number of (*R*)-[^11^C]PK11195 studies remained stable (6 ± 3 per year), but was surpassed by the total number of challenger studies for the last 6 years. In total, 3914 patients underwent a TSPO PET scan, and 47% (1851 patients) received (*R*)-[^11^C]PK11195. The 2 main challengers were [^11^C]PBR28 (24%—938 patients) and [^18^F]FEPPA (11%—429 patients). Only one-in-ten patients (11%—447) underwent 2 TSPO scans, among whom 40 (1%) were scanned with 2 different TSPO radiotracers.

**Conclusions:**

Generally, challengers confirmed disease-specific initial (*R*)-[^11^C]PK11195 findings. However, while their better signal-to-noise ratio seems particularly useful in diseases with moderate and widespread neuroinflammation, most challengers present an allelic-dependent (Ala147Thr polymorphism) TSPO binding and genetic stratification is hindering their clinical implementation. As new challengers, insensitive to TSPO human polymorphism, are about to enter clinical evaluation, we propose this systematic review to be regularly updated (living review).

## Introduction

About 40 years ago, PK11195 [1-(2-chlorophenyl)-N-methyl-N-(1-methylpropyl)-3-isoquinolinecarboxamide] was the first ligand chemically unrelated to benzodiazepines, to label what was called at this time the *peripheral benzodiazepine receptor* (or *omega-3 binding site*) [1]. These terminologies arose from its pharmacology (high affinity for some benzodiazepines) and its preferential expression in peripheral tissues (myocardium, kidney and adrenals) [1, 2]. Now known as the translocator protein 18 kDa (TSPO) [3], this receptor was rapidly identified as over-expressed in brain lesions and thought to be a good marker of neurodegeneration notably in models of brain ischaemia [4, 5]. Once radiolabelled [6], one of the first application of [^11^C]PK11195 for Positron Emission Tomography (PET) was to image brain tumours [7, 8]. However, it was only in the mid- to late 1990’s that increased level of radiolabelled PK11195 *R*-enantiomer binding to TSPO was proven to be associated with microglial activation in many diseases or animal models [9–12]. Though the wide potential of neuroinflammation imaging with (*R*)-[^11^C]PK11195 (correct nomenclature according to latest consensus guidelines [13] and IUPAC rules [14]) was recognized in the early 2000’s [15], intrinsic limitations of this radiotracer (limited brain entry, poor signal-to-noise ratio, plasma protein binding) soon led to the development of so-called “second-generation” TSPO radioligands [16].

In 2008, we published the first systematic review of these “(*R*)-[^11^C]PK11195 challengers”, and listed 45 radiolabelled candidates [17], a number that has now reached more than 60 radiotracers belonging to various chemical classes [18]. However, at that time, most of these radioligands underwent only preclinical evaluation, and clinical evaluation of [^11^C]PK11195 challengers was in its infancy: only 4 tracers had been engaged in a first-in-man study, and 2 reports involved patients ([^11^C]DAA1106 in Alzheimer’s disease [19]; [^11^C]vinpocetine in multiple sclerosis [20]). Since then, many PET studies with (*R*)-[^11^C]PK11195 challengers have been designed for various pathologies. Here we used a systematic approach to examine the outcome of over a decade of clinical evaluation. Studies performed on healthy subjects and comparison of analysis methods were recently reviewed in detail by Cumming et al*.* [21] and are therefore not covered in this review. In contrast, we here concentrate on clinical studies in patients with defined pathology, performed with (*R*)-[^11^C]PK11195 or challengers, with the aim to answer the following questions:

- Has (*R*)-[^11^C]PK11195 been superseded by challengers in clinical studies?
- What is the scientific added-value brought by these challengers?

As an introductory contribution to this TSPO review series, this scoping review covers the full “scope” of neuroinflammation imaging applications. A scoping review uses a systematic approach to “charting” or “mapping” a broad research field, hence it does not include meta-analysis or assess the strength of evidence across studies [22]. Selected fields and pathologies are discussed in detail in other contributions. Our goals are to *i)* provide a quantitative overview of all clinical TSPO PET studies available so far, *ii)* highlight research gaps and advisable future steps for neuroinflammation imaging to realize its full potential.

## Material and methods

This study followed the PRISMA-S and PRISMA-ScR guidelines (PRISMA extensions for reporting literature Searches in systematic reviews [23] and for Scoping Reviews [24], respectively). Both checklists (available from: http://prisma-statement.org/Extensions/) were filled and are provided as supplemental files. The protocol was not registered. Publications were identified by searching MEDLINE (PubMed) electronic database. The following string search was performed:

“((TSPO OR PBR OR (peripheral benzodiazepine receptor[MeSH Terms])) OR (peripheral AND benzodiazepine) OR ("microglial activation") OR ("microglia activation")) AND (positron emission tomography[MeSH Terms] OR PET OR positron) NOT review[pt]”

with i) selection of the “Species = Humans” PubMed limit filter, and ii) final date limit of 2020/12/31. The search was not restricted to interventional studies, and retrieved 433 items (last run on May 2021). In addition to these records, were added: i) 36 recent records without MeSH terms assignment (therefore escaping the Humans filter of Pubmed); ii) 25 records identified through manual full-text assessment which were eligible but escaped the Pubmed search (because of vague description like “macrophage imaging” or “activated glia” and lack of subsequent PBR/TSPO assignment in MeSH terms).

The following criteria were used for inclusion or exclusion:

- Inclusion of any TSPO PET study performed on patients with a defined pathology (CNS or other);
- Exclusion of TSPO PET reports performed only on healthy subjects (first-in-man, dosimetry, quantification methods) or including patients without pathology being studied;
- Exclusion of SPECT studies.

One author (FC) screened the titles and the abstracts for eligibility. The resulting bibliographic database is available as a public, closed membership, Zotero group library (anyone can view, only admins can edit):

### https://www.zotero.org/groups/2578974/living_systematic_review_on_tspo_pet/library.

Two authors (FC and GB) assessed all publications of potential relevance for inclusion and extracted data in a calibrated form (discussed and defined prior to filling). The data extraction form is provided as supplemental material (xls file) and contains the following information for each item: PMID, pathology (coded as a Zotero tag), radiotracer, number of patients/controls, number of patients scanned more than once (if any), treatment (evaluated through TSPO imaging, if any). In case of a new analysis on previously published cohort, the new record was included but the number of patients was set to null so as not count them twice. In case of new patients added to an existing cohort, the record was included, and only new patients were counted. No quality assessment of studies was performed. To summarize data, pathologies were classified into 9 main groups: neurodegenerative diseases, demyelinating diseases, mental disorders, encephalopathies and viral infections, vascular diseases, traumatic brain injury, neuro-oncology, epilepsy and other systemic inflammatory diseases. This simplified classification was performed for statistical overview only. It does not follow a strict methodology but is based on varying criteria such as clinical spectrum, underlying (neuro)pathology or genetic changes. The authors recognize this classification may be questioned or changed according to recent and future knowledge on specific pathologies. All authors reviewed the selected publications and summarised the most relevant findings.

## Results

**Fig. 1:**
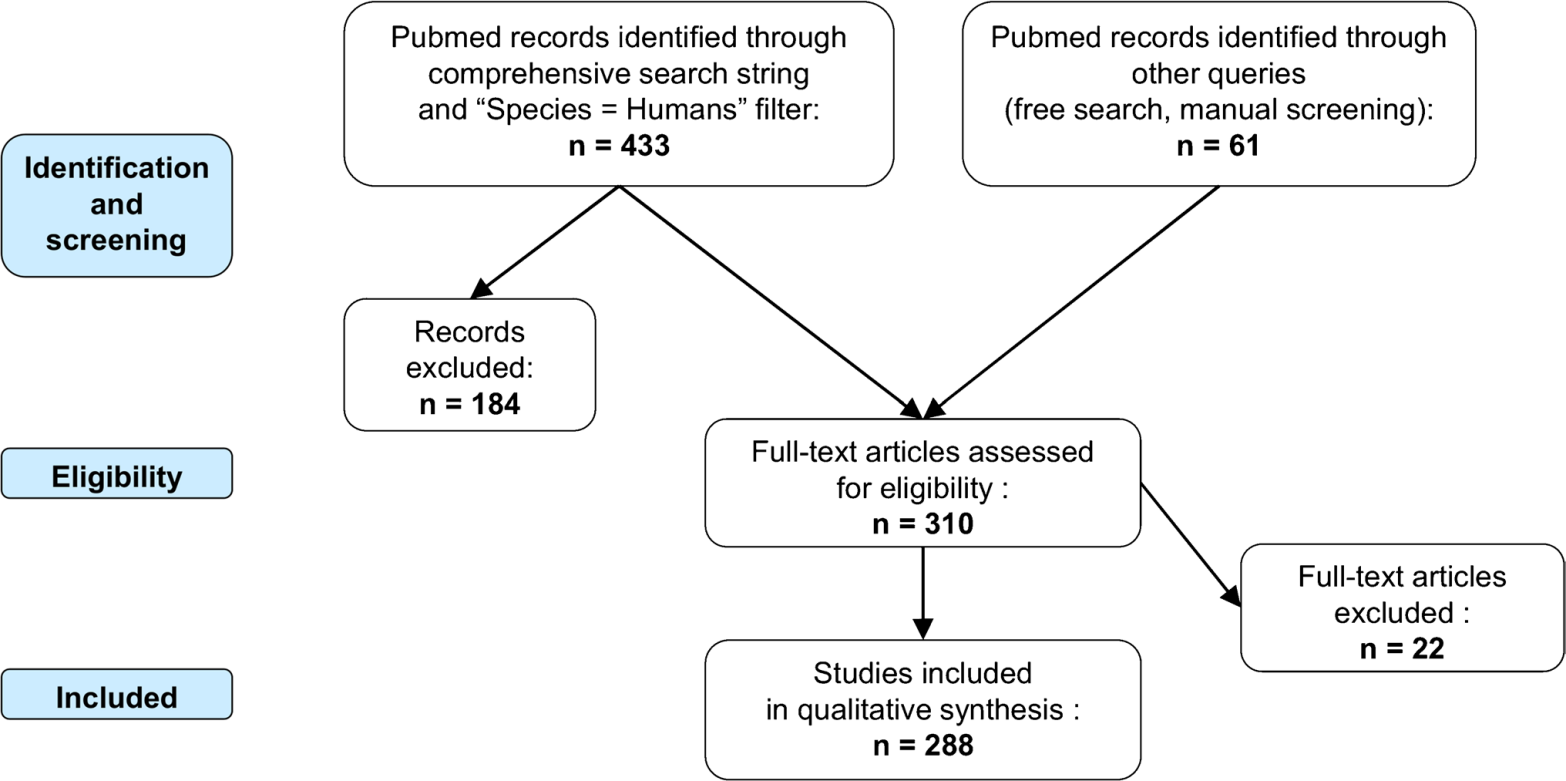
Flow diagram of the study selection process

Figure 1 shows the flow diagram of the study selection process. A total of 288 publications were included, which reported the use of 14 radiotracers ((*R*)-[^11^C]PK11195 and 13 challengers). Approximately half of the studies (142) were conducted with (*R*)-[^11^C]PK11195, and half (152) with the other radiotracers. Of note, only 12 publications (4%) reported the use of more than one radiotracer (identified with the Zotero tag “multiple tracer”). While 6 of them used separate groups of patients, only 40 patients (from 6 studies) received two different radiotracers: (*R*)-[^11^C]PK11195 vs [^11^C]vinpocetine [20, 25]; (*R*)-[^11^C]PK11195 vs [^18^F]GE-180 [26]; (*R*)-[^11^C]PK11195 vs [^18^F]DPA-714 and [^11^C]DPA-713 vs [^18^F]DPA-714 [27]; [^11^C]DPA-713 vs [^11^C]PBR28 [28]; [^11^C]PBR28 vs [^18^F]PBR06 [29].

**Fig. 2:**
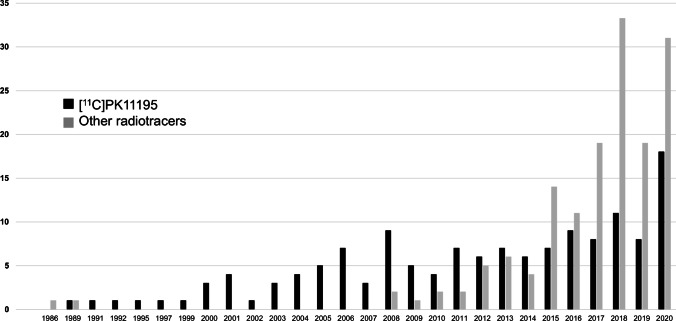
Histogram depicting the number of new studies per year performed with (*R*)-[^11^C]PK11195 or all other “challenger” radiotracers

The number of publications using (*R*)-[^11^C]PK11195 has been keeping a rather stable level from the last 20 years (mean ± SD = 6 ± 3 reports per year over the period 2000–2019). Of note, 2020 results may be biased because the PubMed search with a date limit of 2020/12/31 includes “epub ahead of print” items which will receive a definite date of publication during 2021. As shown in Fig. 2, the annual number of publications with challengers reached the level of (*R*)-[^11^C]PK11195 in 2012 (≥ 5 studies each) and has now surpassed the number of (*R*)-[^11^C]PK11195 studies for the last 6 years (2015–2020).

**Fig. 3:**
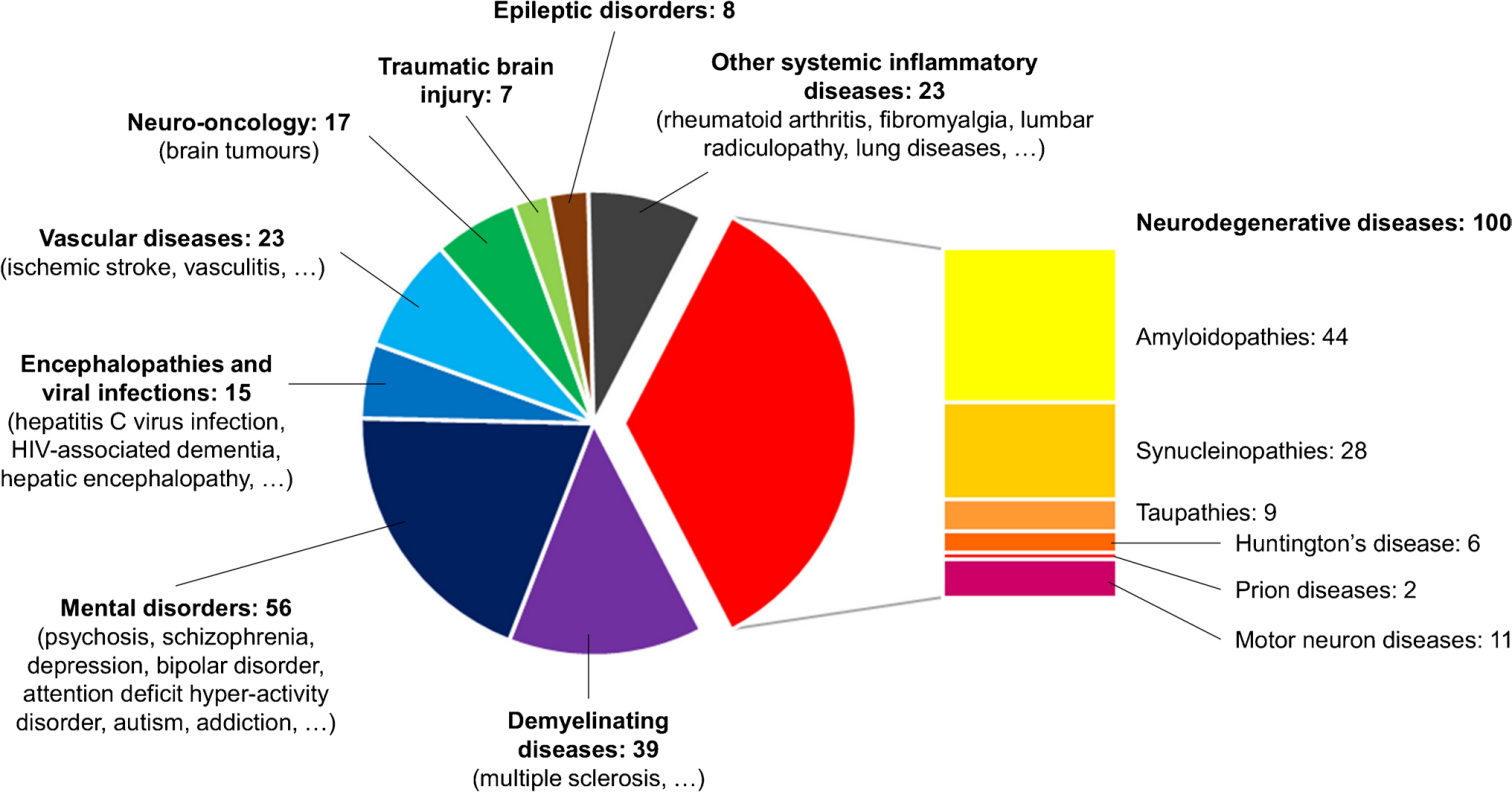
Pie chart showing diseases explored with TSPO PET, with respective number of publications (288 in total)

**Fig. 4:**
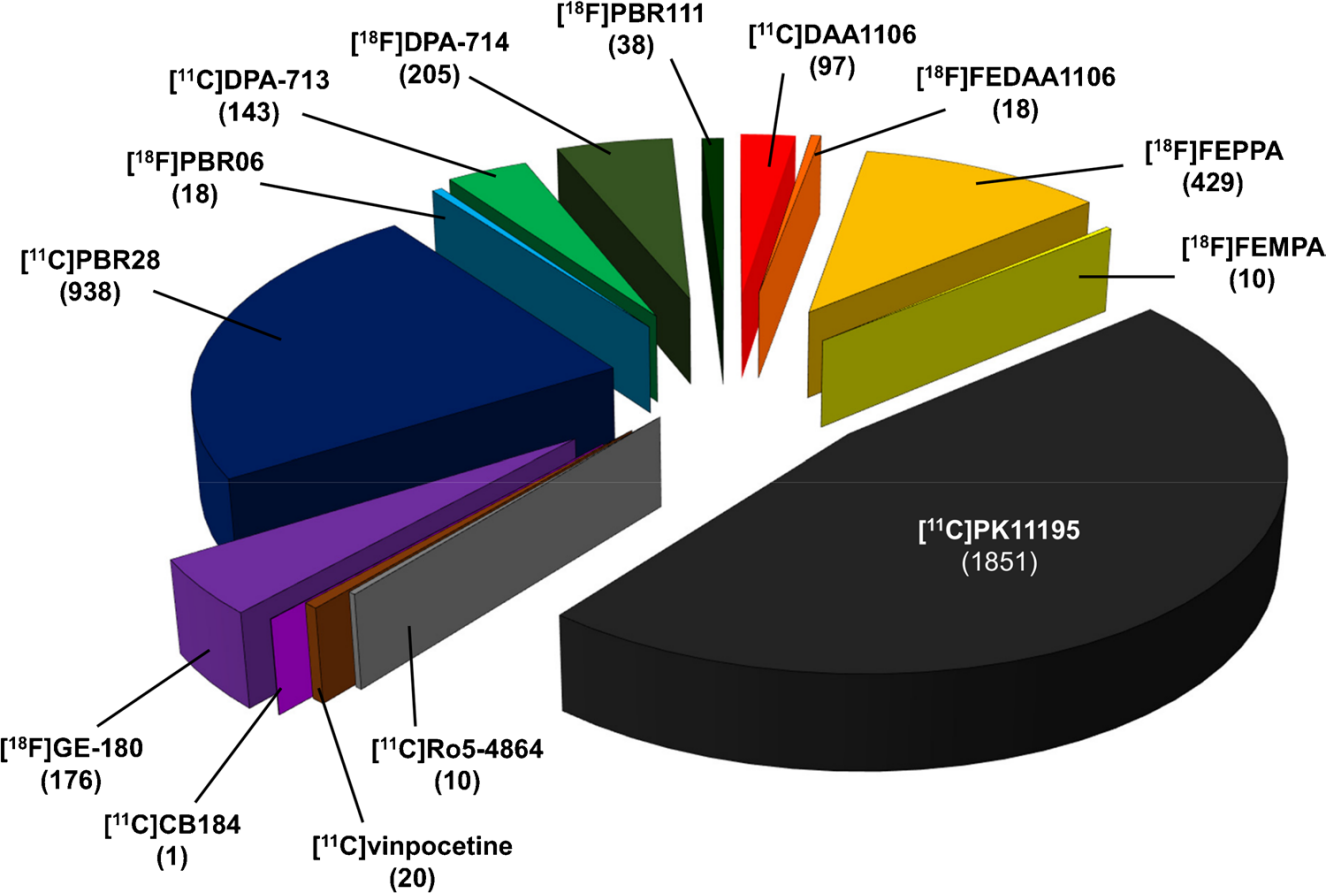
Pie chart showing the number of patients who underwent TSPO PET, according to radiotracers (3954 in total, including the 40 patients who received 2 different radiotracers)

An impressive variety of diseases have been explored: nearly 50 tags were used to classify all pathological conditions on Zotero (according to their description in included publications), which were gathered into 9 main groups (Fig. 3). The publications can be split into 3 thirds: one third for the neurodegenerative diseases, one third for mental disorders and demyelinating diseases, and one third for all the remaining diseases including encephalopathies and viral infections, vascular diseases, neuro-oncology, traumatic brain injury, epilepsy and other systemic inflammatory diseases. Imaging was not restricted to brain, as peripheral imaging was reported in 15 publications (vascular: 4; articular and spinal cord: 9; lung: 2). A summary from data extracted in each main group of pathologies is provided in Table 1. At individual level, a total of 3914 patients underwent a TSPO PET scan, 47% of those (1851 patients) received (*R*)-[^11^C]PK11195. Of the 13 challengers used, 24% (938 patients) had [^11^C]PBR28, 11% (429 patients) had [^18^F]FEPPA and 5% (205) had [^18^F]DPA-714 (Fig. 4). These numbers take in account the re-use of existing experimental groups or cohorts for new analyses (but might be slightly overestimated if new publications do not correctly acknowledge already described datasets). However, it does not take into account healthy controls – a number much more difficult to estimate because of frequent re-use of existing and poorly described cohorts or database. Overall, one in ten patients (11%, 447 patients) underwent a second TSPO scan, whether for a test–retest or longitudinal follow-up with the same radiotracer, or head-to-head comparison between two different radiotracers.

**Table. 1:** Pathologies studied with TSPO PET. The total number of studies (and total number of patients included) is reported for [^11^C]PK11195, for challenger radiotracers altogether, and for the one mainly used (with at least 2 studies). Test–retest, longitudinal follow-up (with the same radiotracer), or head-to-head comparison (with different radiotracers) is specified as the number of studies and number of patients with more than one PET exam/patient

Group	Pathologies (Zotero tags)	Studies with [^11^C]PK11195 radiotracer		Studies with challenger radiotracer		Main challenger radiotracer used
		Nb ofstudies (patients)	Nb of studies (patients) with > 1 PET exam/patient	Nb ofstudies (patients)	Nb of studies (patients) with > 1 PET exam/patient	Nb studies (patients)
NDD	Amyloidopathies (MCI/AD)	23 (406)	3 (54)	26 (384)	2 (38)	[^11^C]PBR28: 11 (210)
	Synucleinopathies (PD(D)/DLB/IRBD/MSA)	20 (218)	4 (21)	8 (139)	2 (40)	[^18^F]FEPPA: 4 (59)
	Tauopathies (FTD/CBD/PSP)	6 (31)	1 (2)	3 (51)	-	-
	Motor neuron diseases (ALS, PLS)	3 (23)	-	8 (144)	1 (10)	[^11^C]PBR28: 5 (124)
	Others (HD, CJD, FFI)	7 (82)	-	1 (8)	-	-
DD	Multiple Sclerosis (MS)	19 (316)	6 (72)	20 (213)	7 (39)	[^11^C]PBR28: 8 (79)
	Others (X-ALD)	1 (1)	-	-	-	-
MD	Psychosis/Schizophrenia (P/S)	7 (152)	-	17 (202)	1 (14)	[^18^F]FEPPA: 9 (107)
	Depression and other disorders (D/BD/OCD)	5 (85)	-	10 (247)	1 (40)	[^18^F]FEPPA: 7 (195)
	Addiction (Add)	1 (12)	-	10 (172)	-	[^11^C]PBR28: 5 (74)
	Various other neuropsychatric conditions*	3 (73)	-	3 (53)	1 (8)	[^11^C]PBR28: 3 (53)
En	HIV-associated dementia (HAD)	3 (29)	-	5 (60)	-	[^11^C]PBR28: 2 (36)
	Others (HSV, HAM, LD, HCV, HE)	5 (48)	1 (2)	2 (19)	-	-
VD	Ischemic stroke (IS)	11 (79)	6 (34)	5 (30)	2 (14)	[^11^C]vinpocetine: 2 (10)
	Others (V, A, ACS, ICH, SVD)	5 (60)	-	4 (29)	2 (9)	[^18^F]DPA-714: 2 (17)
NO	Brain tumors (BT)	7 (63)	-	11 (94)	1 (1)	[^18^F]GE-180: 5 (68)
TBI	Trauma (T)	3 (18)	-	4 (38)	1 (14)	[^11^C]DPA-713: 3 (23)
Ep	Epileptic syndromes (E)	4 (8)	2 (2)	4 (47)	1 (2)	[^11^C]PBR28: 4 (46)
SID	Rheumatoid arthritis (RA)	4 (72)	1 (3)	5 (42)	2 (11)	[^11^C]PBR28: 3 (21)
	Various other systemic conditions**	8 (75)	1 (5)	7 (112)	1 (15)	[^11^C]PBR28: 6 (102)

*autoimmune neuropsychiatric disorders, Gulf war illness, post-traumatic stress disorder, attention deficit/hyperactivity disorder, autism

**fibromyalgia; complex regional pain syndrome; lumbar radiculopathy (low back pain); systemic lupus erythematosus; lung diseases; psoriasis; seasonal allergy; peripheral nerve injury; spasms

## Discussion on each group of disease

In this section, we briefly comment on the comparative results from (*R*)-[^11^C]PK11195 studies *vs* challenger studies in the most studied pathologies. Not all included studies are cited in the text. Only the online Zotero database (described in Methods), and the extraction form provided as supplemental material) contains all the 288 papers included. When available, we refer to previously published meta-analysis, reviews or commentaries.

### Neurodegenerative diseases

#### Alzheimer’s disease

Alzheimer’s disease (AD), and its prodromal condition, mild cognitive impairment (MCI), is the most studied pathology, with a total of 49 publications and 790 patients. It was also one of the first pathology studied apart from glioma, with initial [^11^C]PK11195 and (*R*)-[^11^C]PK11195 reports in patients published in 1995 [30] and 2001 [31]. Among the 8 challengers that have been used for imaging TSPO in MCI/AD, [^11^C]PBR28 is the mostly used (11 publications, 210 patients). Despite a number of detailed reviews [32–34], no meta-analysis is available yet. Overall recent studies performed with (*R*)-[^11^C]PK11195 [35–38] or [^11^C]PBR28 [39, 40], or [^18^F]DPA-714 [41, 42] highlight neuroinflammation as a chronic and biphasic event accompanying amyloid deposition, with a double-edged sword: microglial activation could first be beneficial while turning detrimental in later stages of the disease. But, despite the long disease duration, only 5 publications (3 with (*R*)-[^11^C]PK11195 [37, 43, 44] and 2 with challengers [39, 42]) performed longitudinal studies, so this warrants further investigation with more follow-up studies needed to better understand the time-course of neuroinflammation in AD. Most recent studies combined Tau and TSPO imaging, performed with (*R*)-[^11^C]PK11195 [37, 45] or challenger radiotracers [46, 47]. From these pioneer studies which used different Tau and TSPO radiotracers, it is however difficult to get a clear picture about the association between these two biomarkers and their respective prognostic value.

While historically, imaging studies with (*R*)-[^11^C]PK11195 have produced relatively variable results, with some reports showing an increase and some other no change in AD patients vs controls, more recent studies with various challengers such as [^11^C]PBR28 [39, 48], [^11^C]DPA-713 [49] and [^18^F]DPA-714 [41, 42] seem to have produced more consistent finding even across tracers. Now it is actually difficult to conclude on whether this is strictly down to these tracers having better binding characteristics – such as lower non-specific binding, and hence better signal to noise ratio – or better categorisation of the patients recruited (through amyloid scan) or other factors such as improved and more consistent modelling of TSPO PET. Nevertheless, it is encouraging to see recent studies with new tracers showing consistently that neuroinflammation is present at various stage of the disease and helping to progress disease understanding through future longitudinal studies using (*R*)-[^11^C]PK11195 challengers [37].

#### Synucleinopathies

Parkinson’s disease (PD) and other synucleinopathies have been studied in 28 publications and 357 patients, which overall points to a widespread involvement of neuroinflammation. Notably, TSPO imaging was used in 3 studies to monitor treatment effects [50–52], including one phase-2 study in PD performed with [^11^C]PBR28. Four challengers ([^18^F]FEPPA [53–55], [^11^C]PBR28 [52, 56], [^11^C]DPA-713 [57] and [^18^F]DPA-714 [58]) were used to image TSPO in synucleinopathies, and all 7 studies involved PD patients. Hence DLB and MSA patients have only been scanned with (*R*)-[^11^C]PK11195 yet. In PD, both (*R*)-[^11^C]PK11195 [59] and [^18^F]FEPPA [54] suggested an increase of TSPO binding when patients displayed amyloid co-pathology. Most of the early studies with (*R*)-[^11^C]PK11195 in PD reported significant, although modest (approx. + 10–25%), increase in microglial activation, mostly located in the basal ganglia [59–64], while Bartels et al*.* reported a non-significant trend in PD patients vs controls [50]. Interestingly, a very recent (*R*)-[^11^C]PK11195 study conducted in at-risk subjects (glucocerebrosidase gene mutation carriers without PD) detected neuroinflammation in brain regions susceptible to Lewy pathology [65].

In other conditions, such as idiopathic rapid-eye-movement sleep behaviour disorder, which patients will ultimately develop a synucleinopathy, Stokholm et al*.* showed an increase in (*R*)-[^11^C]PK11195 BP_ND_ in the left substantia nigra and occipital lobe, but not in the basal ganglia [66] or thalamus [67]. Similarly, Surendranathan et al*.* [68] showed elevated (*R*)-[^11^C]PK11195 BP_ND_ in dementia with Lewy body patients with mild symptoms vs healthy controls, while patients with severe symptoms were not different from healthy controls, thus suggesting that it is an early event. Furthermore, neuroinflammation was found to correlate with white-matter changes (in the same cohort [69]), and with Tau deposition (in a pilot study [70]). In multiple system atrophy, a severe form of synucleinopathy with a Parkinsonian syndrome, Dodel et al*.* showed that (*R*)-[^11^C]PK11195 BP_ND_ was significantly reduced by minocycline treatment although without symptoms improvement [51]. More recently and in the same pathology, Kübler et al*.* have shown a significant increase in (*R*)-[^11^C]PK11195 BP_ND_ in basal ganglia and various cortical regions [71]. In most of these studies using (*R*)-[^11^C]PK11195, the presence of microglial activation seems pretty unanimous in synucleinopathies.

In contrast, in the studies using the (*R*)-[^11^C]PK11195 challenger [^18^F]FEPPA, Koshimori et al*.* [72] and Ghadery et al*.* [53] showed that only the TSPO polymorphism rs6791 had a significant impact on uptake in both healthy controls and PD patients but could not detect any significant difference between controls and PD subjects; results that are in agreement with a more recent study using [^11^C]PBR28 [56]. However, in a subsequent study [54], in which Ghadery et al*.* included an amyloid scan with [^11^C]PIB, they found no significant differences between controls and PD in PIB-negative subjects, whereas PIB-positive PD patients had increased [^18^F]FEPPA *V*_*T*_ in several brain regions, suggesting a potential interaction between Aβ deposition and neuroinflammation in PD. Those studies with [^18^F]FEPPA and [^11^C]PBR28 are in contrast with those obtained with [^11^C]DPA-713 [57] and [^18^F]DPA-714 [58], who showed significant BP_ND_ increases in numerous brain regions in PD patients vs healthy controls, with further increase in a follow-up scan 1 year later [57]. Overall, those studies with (*R*)-[^11^C]PK11195 challengers in synucleinopathies are highlighting the potential issues brought by some tracers whose binding is sensitive to the rs6791 polymorphism in complex diseases with moderate and widespread neuroinflammation, harder to detect than in focal brain injuries. Altogether, these results warrant further investigations to better understand the role and time-course of neuroinflammation in synucleinopathies and to determine which tracers perform the best in such challenging situation.

#### Tauopathies

Fronto-temporal dementia (FTD) was examined only in pilot studies (4 studies with ≤ 5 patients each), performed with (*R*)-[^11^C]PK11195 [73, 74], [^11^C]DAA1106 [75] and [^11^C]PBR28 [76]. In brief, Cagnin et al*.* [73] first showed strong increases in (*R*)-[^11^C]PK11195 BP_ND_ in the putamen, hippocampus and dorsolateral frontal cortex. Bevan-Jones et al*.* [74] later showed similar changes, although in a single patient with microtubule associated protein tau (MAPT) mutation, in the left lateral anterior temporal lobe and bilaterally in the fusiform gyrus. Interestingly, their results suggested that (*R*)-[^11^C]PK11195 PET was better at discriminating MAPT mutation carrier from controls than Tau protein aggregation measured by [^18^F]AV-1451 PET, highlighting an early role for neuroinflammation at the pre-symptomatic stage of the disease. In a more recent study in patients with frontotemporal dementia with parkinsonism linked to chromosome 17 (FTDP-17), Miyoshi et al*.* [75] showed inhomogeneous increases in [^11^C]DAA1106 uptake in brain regions varying from patient to patient (n = 3). Finally, Kim et al*.* [76] showed that [^11^C]PBR-28 *V*_*T*_ were significantly increased in frontal, lateral, temporal, parietal, and occipital cortices of FTLD patients vs controls.

Other taupathies (corticobasal degeneration, progressive supranuclear palsy) were studied with (*R*)-[^11^C]PK11195 (4 publications, 25 patients [77–80]), and in one very recent study with [^18^F]GE-180 (combining 30 CBD and 14 PSP [81]). All studies reported a consensual increased binding in the striatum (CBD and PSP) and cortical areas (CBD > PSP).

#### Huntington’s disease (HD)

All 6 publications available (77 patients in total) but one used (*R*)-[^11^C]PK11195. The single [^11^C]PBR28 study confirmed a marked increase in the striatum of HD patients, and successfully distinguished patients from controls at the individual level upon principal component analysis [82].

#### Prion diseases

A single research group published two (*R*)-[^11^C]PK11195 reports that showed increased TSPO with varying patterns across subtypes of Creutzfeldt-Jakob disease [83] or Fatal Familial Insomnia [84]. There is no report using other radiotracers.

#### Motor neuron diseases

Motor neuron diseases are a group of neurological disorders with progressive loss of motor neurons which includes *amyotrophic lateral sclerosis* and *primary lateral sclerosis*. Of the 11 publications available, most enrolled patients with amyotrophic lateral sclerosis (ALS), but 3 recruited patients with primary lateral sclerosis (PLS). The main challenger was [^11^C]PBR28 (5 publications, 124 patients). All studies corroborated an increase in TSPO radioligand uptake which was not restricted to anatomically relevant motor regions and was related to the clinical phenotype (bulbar onset vs limb onset). In the first study with (*R*)-[^11^C]PK11195, Turner et al*.* [85] showed a significant increase in BP_ND_ in motor regions but also in non-motor regions such as frontal lobe, pons and thalamus, with anatomical pathways between motor cortices and those non-motor brain regions likely to explain these changes. Two studies have been performed with [^18^F]DPA-714 which showed consistent increases in motor cortices of ALS patients vs controls [86, 87]. In a subsequent study, Van Weehaeghe et al*.* [88] pooled data from their own study using [^18^F]DPA-714 and data from a [^11^C]PBR28 study [89] in ALS study and confirmed increases in SUVR for both tracers. This suggests that data from different studies with different TSPO tracers using standardised analysis could be gathered, at least in ALS patients, for larger group analysis. In the 4 studies using [^11^C]PBR28 [89–92], there was overall consensus of significant increases in tracer uptake in ALS patients vs controls, although brain regions affected varied slightly from one study to the another, with no further increase at 6 month in follow-up scans being reported by Alshikho et al. [91]. One recent study focused on familial ALS and recruited symptomatic and asymptomatic carriers (SOD1 mutation cases) [93]. (*R*)-[^11^C]PK11195 was increased in asymptomatic carriers and further increased in symptomatic carriers, in line with the suggested pattern of progression of the pathology, from spinal cord and brainstem to the cortical areas. Hence (*R*)-[^11^C]PK11195 and challenger studies showed overall consistent increases in neuroinflammation in ALS.

### Multiple sclerosis (MS)

MS is the most widely studied pathology after AD, with a total of 39 publications and 529 patients. Despite this large amount of data, no meta-analysis has been conducted yet. However, a narrative review was published in 2018 and included 23 studies [94]. The contribution of TSPO PET in MS was also recently tackled in a “Controversies” section of the Multiple Sclerosis Journal [95]. Importantly, it is the only disease for which a significant number of patients (around 20%) underwent two scans (6 studies with (*R*)-[^11^C]PK11195, 7 with challengers). These included longitudinal studies [96], monitoring treatment effects [97–101], test–retest studies [102–104], but also rarely performed designs like head-to-head comparisons of tracers in the same patients [20, 29], and a blocking study with the non-radioactive TSPO ligand XBD173 to measure non-displaceable binding of radiotracers [105]. Eight challengers have been used in MS, the main one being [^11^C]PBR28 (8 publications, 79 patients). Diffuse neuroinflammation, involving normal-appearing white matter and possibly grey matter, was noted in early reports with (*R*)-[^11^C]PK11195 [106, 107] and more precisely assessed with challengers [108]. The general idea is that TSPO PET unmasks active lesions and thus reveals hidden but ongoing MS pathology not necessarily revealed by MRI. Indeed, TSPO PET pointed out the heterogeneity of gadolinium-negative lesions, thus leading to a PET-derived classification (active/inactive) of chronic lesions or black holes [103, 109]. Diffuse neuroinflammation in normal appearing white-matter and in normal appearing grey matter, was more pronounced in progressive MS than in relapsing–remitting phenotypes [94]. Several studies pointed out the predictive value of TSPO PET ((*R*)-[^11^C]PK11195 [110, 111], [^11^C]PBR28 [112], [^18^F]DPA-714 [113]). Overall, in spite of the complexity brought by the different MS subtypes, results from (*R*)-[^11^C]PK11195 and challenger studies were in accordance. Importantly, both (*R*)-[^11^C]PK11195 and challengers were able to track treatment effects, as shown in studies with Fingolimod, the first oral disease-modifying therapy developed for MS [97, 99].

No other demyelinating condition has been explored to date, except a paediatric case report of *X-linked adrenoleukodystrophy*, scanned with (*R*)-[^11^C]PK11195 [114].

### Mental disorders

#### Psychosis/Schizophrenia (P/S)

This is the most widely studied psychiatric disorder (24 publications, 354 patients), but a single longitudinal study has been reported to date (performed with [^11^C]PBR111 [115]). Importantly, the results in this field have been abundantly discussed in one review published in 2017 (which included 11 studies, performed with (*R*)-[^11^C]PK11195 and challengers [116]), followed by two meta-analysis—one conducted on all TSPO radiotracers (12 studies [117]), and one restricted to challengers (5 studies [118])—which yielded opposite conclusions. The latter found strong evidence of lower levels of TSPO in patients, which could correspond to altered function or lower density [118]. The former found an increase of TSPO PET tracer binding in patients, only when non-displaceable binding potential BP_ND_ (mainly used in (*R*)-[^11^C]PK11195 studies) was used as an outcome measure. When tracer volume of distribution *V*_*T*_ was used (mainly in challenger studies), the difference was absent [117]. The interpretation of these heterogenous results is not straightforward, and various sources of biological variation have been put forward (medication, sub-group differences), in addition of PET methodological considerations [119]. While specific recommendations for future TSPO studies in P/S have been proposed [119], including the need to perform longitudinal studies, others questioned the specificity of TSPO (through post-mortem evaluation of TSPO densities) and call for new neuroinflammation targets to be explored [120].

#### Depression

Patients with major depressive disorder were enrolled in 11 studies, performed with (*R*)-[^11^C](*R*)PK11195 [121–123] or [^18^F]FEPPA [124–129] or [^11^C]PBR28 [130, 131]. Six of them were included in a meta-analyse [132], showing a most-pronounced increase in anterior cingulate cortex and hippocampus. Overall, the studies pointed out the potential involvement of two other regions, namely the frontal cortex and the insula. A cross-sectional study published by Setiawan et al*.* in the Lancet Psychiatry convincingly demonstrated using [^18^F]FEPPA that *V*_*T*_ was significantly higher in patients with major depressive disorder untreated for a long period compared to those untreated for a short period of time [127]. Thus, they suggested that the increase of microglial activation over the curse of the disease was disrupted by antidepressant treatment. Two other [^18^F]FEPPA studies were used in conjunction with non-pharmacological (psychotherapy [126]) and pharmacological (celecoxib [128]) treatments. These studies respectively reported that when cognitive-behavioural therapy efficiently reduced the symptoms, it also reduced [^18^F]FEPPA *V*_*T*_ [126], and that Celecoxib therapy was efficient only in patients with high [^18^F]FEPPA *V*_*T*_ values in frontal cortex and anterior cingulate cortex [128]. Though [^18^F]FEPPA was successfully used in several large studies, the only study performed with (*R*)-[^11^C]PK11195 (along with a pilot report [121]) revealed the same results in the anterior cingulate cortex (with a relatively high effect size), whereas the effects were less pronounced in the frontal cortex and the insula [122]. Importantly, these conclusions have been very recently confirmed in a new, large cohort of 51 patients scanned with (*R*)-[^11^C]PK11195 [123, 133].

One [^11^C]PBR28 study enrolled healthy subjects exposed to psychosocial risk factors (and therefore at risk of *depression* or *psychosis*) and found no difference with an unexposed, group [134].

#### Addiction

TSPO PET studies on addiction disorders include a single (*R*)-[^11^C]PK11195 report published in 2008 [135], and 10 more recent publications performed with 3 different challengers ([^11^C]PBR28 [136–140], [^11^C]DAA1106 [141–143], [^18^F]FEPPA [144, 145]). Results were highly dependent on *i)* addiction subtypes (*i.e.* methamphetamines users, alcohol-dependent, cigarette smokers, cannabis smokers, …), *ii)* patient state (active user vs abstinent), and *iii)* experimental design (*e.g.* time between last exposition and PET scanning). Longitudinal data are awaited to understand the relationship between chronic use and TSPO density.

#### Other mental disorders

Sparse data are available for various other psychiatric illnesses: *bipolar disorder* (2 publications using the same cohort scanned with (*R*)-[^11^C]PK11195 [146, 147]); *obsessive–compulsive disorder* (2 publications using [^18^F]FEPPA [129, 148]); a single (*R*)-[^11^C]PK11195 study on adults with *attention deficit/hyperactivity disorder* [149]; single studies on *post-traumatic stress disorder* [150] and *Gulf war illness* [151], both using [^11^C]PBR28; one (*R*)-[^11^C]PK11195 publication dedicated to children with autoimmune neuropsychiatric disorders (associated with streptococcal infection, and Tourette syndrome) [152]; and 2 studies ((*R*)-[^11^C]PK11195 [153] and [^11^C]PBR28 [154]) on young autistic adults, which yielded opposite results. While the (*R*)-[^11^C]PK11195 study found higher BP values in multiples regions and especially in the cerebellum, the [^11^C]PBR28 study reported decreased SUVR in several regions and no region with higher SUVR than controls. The (*R*)-[^11^C]PK11195 study used dynamic scans, and, unusually the cerebellum from controls was used as reference region for the simplified reference tissue model in the patients. The [^11^C]PBR28 study used static scans and whole brain mean normalisation for SUVR calculations. Decreased TSPO densities are difficult to interpret: it might be related to a physiological role of TSPO, and could in the case of autism reveal mitochondrial dysfunction, as suggested by the authors [154].

### 4.4 Encephalopathies and viral infections

*HIV-associated dementia.* It has been hypothesized that microglial activation could be responsible for the cognitive impairments found in some HIV-positive patients despite effective antiviral treatment. Initial (*R*)-[^11^C]PK11195 studies yielded inconsistent results, with an increase of TSPO radiotracer binding being detected [155, 156] or not [157]. Similarly heterogeneous results were obtained with [^11^C]PBR28 [158, 159], so no conclusion can be drawn on the role of neuroinflammation in AIDS, as recently reviewed [160].

Others viral infections explored included: *Lyme disease* with [^11^C]DPA-713 [161]); *Herpes simplex encephalitis* with (*R*)-[^11^C]PK11195 [162]; *hepatitis C* with (*R*)-[^11^C]PK11195 [163, 164] and *T-lymphotropic virus Type 1* with [^11^C]PBR28 on associated myelopathy [165].

Finally, two [^11^C]PK11195 reports in *hepatic encephalitis* in (< 10) cirrhotic patients yielded different results, one (using the *R*-enantiomer) finding increased uptake in comparison to controls [166], but not the other (using the racemic radiotracer) [167].

### Ischemic stroke and other vascular diseases

Most stroke studies were performed with (*R*)-[^11^C]PK11195 (11 publications on ischemic stroke, one publication on haemorrhagic stroke [168], 84 patients in total). Ten stroke patients were scanned with [^11^C]vinpocetine, among which 4 back-to-back with (*R*)-[^11^C]PK11195 [25, 169] and 9 patients with [^18^F]DPA-714 [170]. In addition, one recent study directly compared (*R*)-[^11^C]PK11195 and [^18^F]GE-180 in the same 10 patients at the sub-acute stage (< 1 month), and discarded the use of the latter because of poor brain uptake and strong contribution of vascular signals [26]. Few patients (< 20 in total) were scanned at the chronic stage, up to 5–6 months after stroke onset [171, 172]. The late TSPO changes in the area of the primary lesion, but also in areas distant from the primary lesion site, probably deserve to be explored further, as they might play a role in either recovery or indicate long-term distal retrograde degeneration.

One recent study focused on small vessel disease and highlighted increased, mostly vascular, [^11^C]PBR28 binding in white matter hyperintensities [173].

Peripheral imaging was performed to study *atheroma* (one (*R*)-[^11^C]PK11195 report [174]), *acute coronary syndrome* (one [^18^F]DPA-714 report [175]), and *vasculitis* (two (*R*)-[^11^C]PK11195 reports [176, 177]). The latter pathology was recently imaged, this time at the brain level, with [^18^F]DPA-714 [178].

### Traumatic Brain Injury (TBI)

Relatively few studies investigated the long-term neuroinflammation after trauma (3 (*R*)-[^11^C]PK11195 publications [179–181], 4 with challengers [182–185]). Severity varied greatly with aetiology, making inter-study comparison perilous. Overall, studies agreed on the fact that TBI triggered a chronic inflammatory response particularly in subcortical regions, with a specific involvement of the thalamus. [^11^C]DPA-713 was used in the specific context of chronic traumatic encephalopathy (CTE), a recently diagnosed disease described in American football players [182, 183]. Localized brain injury and repair, indicated by increased [^11^C]DPA-713 uptake, may be linked to history of football-related repeated traumatic brain injuries, although further studies are needed to determine whether TSPO signals in CTE are related to later onset of neuropsychiatric symptoms or altered functional connectivity [185]. Interestingly, in an pilot clinical trial involving patients with moderate-to-severe TBI, minocycline treatment reduced chronic microglial activation as assessed with [^11^C]PBR28, but increased a plasma marker of neurodegeneration [184]. The lack of similar study with (*R*)-[^11^C]PK11195 precludes any formal comparison although one study revealed a thalamic TSPO increase up to 17 years after TBI [180].

### Neuro-oncology

Brain tumours were studied in 7 publications with (*R*)-[^11^C]PK11195 and 11 publications with challengers. This includes the oldest reports of TSPO imaging in humans, performed with [^11^C]Ro5-4864 [8, 186], a radiotracer which was abandoned for [^11^C]PK11195. Six other publications are case reports. So in fact this field remains relatively unexplored, and longitudinal studies are lacking (2 [^11^C]PBR28 scans were performed in a single case report of astrocytoma [187]). The different studies, with either (*R*)-[^11^C]PK11195 or challengers, reported specific binding of TSPO radioligands by tumour cells thereby pointing to the difficult discrimination between reactive neuroinflammation and tumour development itself [188]. This is clearly illustrated by studies using the challengers [^18^F]GE-180 [189] and [^18^F]DPA-714 [190] along with amino-acid radiotracer ([^18^F]Fluoro-ethyl-tyrosine), which showed diverging spatial extent of the different radiotracers. These results highlighted the heterogeneity of the immune tumour microenvironment on one hand, and on the other hand, the fact that neuroinflammation may not overlap with tumour proliferation. As another example of this caveat, neuroinflammation in the context of brain metastases was recently reported in 5 cases of non-small cells lung carcinoma and melanoma brain metastases [191], but [^11^C]PBR28, in contrast to [^11^C]methionine, could not differentiate metastatic tumour recurrence from neuroinflammation-induced radiation necrosis.

### Epileptic disorders

*Epileptic syndromes* may have a variety of aetiologies. Some cases of Rasmussen’s encephalitis and hippocampal sclerosis were studied with (*R*)-[^11^C]PK11195 [12]. Few other studies were performed in temporal lobe epilepsy [28, 192], neurocysticercosis [193], and neocortical epilepsy [194] using [^11^C]PBR28 or [^11^C]DPA-713. Apart from the neurocysticercosis cases, in which acute perilesional oedema caused the inflammation revealed by TSPO uptake, all studies agreed on a clear asymmetry of PET signals with increased uptake in the hemisphere ipsilateral to the seizure foci, despite frequent involvement of bilateral regions. Interestingly, the computed asymmetry index was higher in patients suffering from mesial temporal sclerosis. Considering the low number of studies, and the lack of direct head-to-head comparison between (*R*)-[^11^C]PK11195 and challengers, it is difficult to conclude on the added value of such tracers. However, the pioneer (*R*)-[^11^C]PK11195 study by Banati et al*.* [12] did not find a lateralized increase of binding in 3 patients with temporal lobe epilepsy (TLE), in contrast to Rasmussen’s encephalitis [12] and refractory epilepsy [195] imaged with (*R*)-[^11^C]PK11195, and in contrast to further [^11^C]PBR28 [28, 192–194] or [^11^C]DPA-713 [28] studies in TLE. While this could be due to low seizure frequency in this clinically-stable patients, one might also hypothesize that [^11^C]PK11195 lacked sensitivity to catch a modest TSPO increase in epilepsy.

### Systemic inflammatory diseases

#### Rheumatoid arthritis (RA)

TSPO PET allowed the detection of active clinical but also subclinical synovitis in joints (knees, wrists, hands). After 3 initials (*R*)-[^11^C]PK11195 studies which used a short static scan and scoring scale to assess overall uptake on multiple joints [196–198], there was a renewed interest in this pathology driven by the use of various challengers and SUV quantification [27, 199–201]. In one study, head-to-head comparisons of 3 radiotracers provided a clear demonstration of increased target-to-background ration with [^11^C]DPA-713 [27]. Successful blocking of [^11^C]PBR28 binding with XBD173 was reported very recently [201]. Finally, the involvement of the brain immune system in RA was recently explored with [^11^C]PBR28 [202], but no differences in TSPO binding were detected in RA vs controls.

#### Other systemic inflammatory diseases

Several other inflammatory diseases were explored in pioneer studies: *fibromyalgia* (2 publications, with (*R*)-[^11^C]PK11195 [203] and [^11^C]PBR28 [204]); *lumbar radiculopathy* usually referred as *chronic low back pain* (4 publications with [^11^C]PBR28 [205–208]); *type 1 complex regional pain syndrome* (2 publications on the same cohort with (*R*)-[^11^C]PK11195 [209, 210]); *lung diseases* (2 publications with (*R*)-[^11^C]PK11195 [211, 212]); as well as *systemic lupus erythematosus* [213], *psoriasis* [214], *seasonal allergy* [215], *peripheral nerve injury* [216], *spasms* [217].

## General discussion

Many excellent narrative reviews on TSPO PET have been published these last few years [218–221], and dozens of critical literature reviews covered more or less extensively this topic in the main diseases with neuroinflammatory component [33], like Alzheimer’s disease [34, 222] or multiple sclerosis [94]. We here performed the first systematic search and review on clinical applications of TSPO PET. By assessing the total number of studies and patients (and taking in account the re-use of cohorts), we provided a quantitative overview of the wide field of neuroinflammation imaging. This is a timely achievement to question the impact of (*R*)-[^11^C]PK11195 challengers as most studies with these co-called second-generation tracers were performed in the last 5 years (Fig. 2).

In total, 13 challengers were used to scan patients, 4 of which had a marginal use. First, [^11^C]Ro5-4864 was developed along with [^11^C]PK11195 and was abandoned after 2 pilot neuro-oncology studies in the 80’ [8, 186]. Second, a pathophysiological uptake of [^11^C]CB184 was described in a single case report of cerebellar ataxia associated with HIV infection [223]. Finally, the use of [^18^F]FEMPA and [^18^F]FEDAA1106was restricted to one and two pilot studies respectively, all performed at the Karolinska Institute (Stockholm, Sweden) [224–226]. Two other radiotracers were used in several studies or pathological conditions, but emanated from a single research centre. This is the case of [^18^F]PBR06 (3 publications in MS patients [29, 227, 228] performed at Harvard Medical School, Boston, USA) and [^11^C]vinpocetine (4 publications in AD [229], MS [20] and stroke [25, 169] patients, all performed at the Karolinska Institute. In the case of [^18^F]PBR111, two independent research groups conducted studies in multiple sclerosis [103, 230, 231] (Imperial College London, UK) and schizophrenia/psychosis [115, 232] (University of Antwerp, Belgium). Among the 6 remaining challengers, 2 were restricted to specific groups of pathologies: [^11^C]DAA1106 and [^18^F]FEPPA were used only in neurodegenerative diseases and mental disorders ([^11^C]DAA1106: 20 and 91 patients respectively; [^18^F]FEPPA: 77 and 337 patients respectively). [^18^F]GE-180, initially mainly used in patients with multiple sclerosis (45 patients) and brain tumour (68 patients), was recently tested in other pathologies (tauopathies [81], rheumatoid arthritis [200] and stroke [26]). However, [^18^F]GE-180 was shown to have slow/poor brain uptake in human [233], and the consequences on quantification and modelling have abundantly debated [234–236]. Finally, only [^11^C]PBR28 covered the 9 disease groups (938 patients in total). [^11^C]DPA-713 (7 disease groups) and [^18^F]DPA-714 (5 disease groups) were also widely used but cumulated a much lower number of patients (143 and 205 respectively).

Interestingly the main challenger, [^11^C]PBR28, shares with (*R*)-[^11^C]PK11195 the disadvantage of a carbon-11 labelling, thereby limiting its availability. Hence to date, fluorinated radiotracers for TSPO, like [^18^F]DPA-714 and [^18^F]FEPPA, remained under-used in comparison to [^11^C]PBR28 and (*R*)-[^11^C]PK11195. Despite displaying higher affinity and specific binding than (*R*)-[^11^C]PK11195, [^11^C]PBR28 has the disadvantage of being highly sensitive to the human Ala147Thr polymorphism [237] because of its strong affinity for the high-affinity binding site, an issue that might discourage research groups working with (*R*)-[^11^C]PK11195 from switching to another radiotracer. This human polymorphism, along with specific blood–brain characteristics different from animals, as highlighted in the case of [^18^F]GE-180 in stroke [26, 238], further emphasizes the need for rapid translation from animal model to human to thoroughly validate radiotracers. Despite the increasing number of challengers entering clinical evaluation, (*R*)-[^11^C]PK11195 studies have been constantly accumulating over the last twenty years, with no trend of disinterest. This is in itself a sign that this historical TSPO tracer has not been outclassed by any of the challengers yet although the number of studies using challengers per annum has now surpassed the number of (*R*)-[^11^C]PK11195 studies. One straightforward reason is that (*R*)-[^11^C]PK11195 is unsensitive to the TSPO polymorphism. However this situation is expected to change in the near future as some of the newly developed (*R*)-[^11^C]PK11195 challengers, sometimes called third-generation tracers, currently being evaluated in animal models and in healthy human subjects, were successfully tested as insensitive or less sensitive to TSPO polymorphism in humans: [^18^F]LW223 [239]; [^18^F]CB251 [240]; [^11^C]ER176 [241]. The latter was suggested to have very favourable properties in healthy subjects, being sensitive enough to detect specific binding in low-affinity binders, with little influence of radiometabolites [242]. Therefore, we can anticipate that the coming years will be crucial for TSPO imaging as these new challengers will be tested in patients.

Overall, the multiplicity of tracers in preclinical studies has translated in multiple, clinical studies with various radiotracers. Owing to the inherent difficulties and debates about the modelling of TSPO PET data, it is difficult to draw conclusions and identify an area with a clear added-value of challengers, despite their predicted superiority [243]. In this review we could however identify the following pathologies in which challengers were efficient in strengthening initials results suggested by (*R*)-[^11^C]PK11195 studies and/or in renewing an interest in TSPO imaging: Alzheimer’s and Parkinson’s disease, amyotrophic lateral sclerosis, multiple sclerosis, epilepsy, rheumatoid arthritis. In most cases, challengers brought confirmation of (*R*)-[^11^C]PK11195 results, rather than true novelty. One particular case is the field of psychiatric diseases, in which the number of studies performed with challengers is notably higher than the number of (*R*)-[^11^C]PK11195 studies (40 vs 16). This led to concordant results (in the case of depression), or apparently conflicting results (in the case of psychosis/schizophrenia). We did not identify any proper replication study (similar experimental groups, similar quantification methods) performed with (*R*)-[^11^C]PK11195 and another radiotracer. This point deserves peculiar attention as replication studies would be a clear asset to investigate the potential benefits of challenger TSPO PET radiotracers over (*R*)-[^11^C]PK11195. More generally, replication studies are cruelly lacking in biomedical sciences, and sciences agencies start to handle this problem with dedicated funds [244]. Hence, cornerstone studies on TSPO PET imaging would definitely benefit from replication. Finally, of particular importance are rare but highly-informative reports such as i) blocking studies with newly approved TSPO ligands like XBD173 [105, 201, 242], ii) double-tracer studies (12 records).

A striking feature of this systematic review is the very weak proportion of longitudinal studies. Our 11% count pooled test–retest studies and multiple-tracer studies so the real proportion of patients who underwent a follow-up scan is even below. This is paradoxical as TSPO PET is often promoted for its ability to dynamically track inflammation changes over time. However, this weakness may be justified by the significant proportion of studies in which TSPO PET scan was associated with another radiotracer (e.g. [^11^C]PIB in Alzheimer, [^18^F]FET in tumours) hence adding a dosimetry issue for the management of longitudinal studies. Another reason is probably the rarely-studied, high (and method-dependent) intra-subject variability which complicates longitudinal monitoring of TSPO changes [102, 245–247]. This last point, together with the poor specificity of TSPO expression (whether it be for a cell type or a type of activation [120, 248]) might ultimately questions the utility of developing so many tracers for the very same target. In that view, back-to-back comparison between TSPO and another inflammatory target (as performed in the case of P2X7 [87]) is of utmost importance for the validation of new clinically-relevant radiotracers. As metabolic changes of senescent myeloid cells are being deciphered [249], new original targets may help to identify and ultimately prevent maladaptive pro-inflammatory responses.

We propose this contribution as the first version of a living review on TSPO PET in the clinics. Living reviews are regularly updated to reflect emerging trends or evidence [250]. With version 2 planned at the beginning of year 2023 (covering 2021–22 years), we will address limitations of the current work and provide timely information for the design of future studies, that is:

- multi-database search to include, in addition to PubMed records, conference proceedings, preprint publications and registered trials, with the goal to better catch ongoing research, as new challengers are to enter the arena;
- include normal aging studies and pharmacological challenges (LPS, XBD-173) performed in healthy controls, which were excluded at this stage but bring important information;
- provide online infographic summaries of clinical TSPO PET studies, possibly interactive, based on the figures presented here.

## Data Availability

All data generated or analysed during this study are included in this published article and its supplementary information files.

## Abbreviations

NDD: Neurodegenerative diseases
MCI: mild cognitive impairment
AD: Alzheimer’s disease
PD(D): Parkinson’s disease (with dementia)
DLB: dementia with Lewy Bodies
IRBD: idiopathic rapid-eye-movement sleep behaviour disorder
MSA: multiple system atrophy
FTD: frontotemporal dementia
CBD: corticobasal degeneration
PSP: progressive supranuclear palsy
ALS: amyotrophic lateral sclerosis
PLS: primary lateral sclerosis
HD: Huntington’s disease
CJD: Creutzfeldt-Jakob disease
FFI: Fatal Familial Insomnia
DD: Demyelinating Diseases
MS: Multiple sclerosis
X-ALD: X-linked adrenoleukodystrophy
MD: Mental Disorders
P/S: Psychosis/Schizophrenia
D/BD/OCD: Depression/Bipolar disorder/Obsessive–Compulsive Disorder
Add: Addiction
En: Encephalopathies and viral infections
HAD: HIV-associated dementia
HSV: Herpes encephalitis
HAM: HTL Virus Type 1–associated myelopathy
LD: Lyme disease
HCV: hepatitis C virus infection
HE: hepatic encephalopathy
VD: Vascular Diseases
IS: ischemic stroke
V: vasculitis
A: atherosclerosis
ACS: acute coronary syndrome
ICH: intracerebral haemorrhage
SVD: small vessel disease
NO: NeuroOncology
BT: Brain tumors
TBI: Traumatic Brain Injury
T: trauma
Ep: Epileptic disorders
E: Epileptic syndromes
SID: other Systemic Inflammatory Diseases
RA: rheumatoid arthritis ^**^
